# Salivary Characteristics, Individual Casual Parameters, and Their Relationships with the Significant Caries Index among Korean Children Aged 12 Years

**DOI:** 10.3390/ijerph18063118

**Published:** 2021-03-18

**Authors:** Jae-Hwan Kim, Mi-Ah Kim, Yong Kwon Chae, Ok Hyung Nam

**Affiliations:** 1 Department of Pediatric Dentistry, School of Dentistry, Jeonbuk National University, Jeonju 54896, Korea; jhbcss@hanmail.net; 2 Department of Conservative Dentistry, School of Dentistry and Institute of Oral Bioscience, Jeonbuk National University, Jeonju 54896, Korea; miah2018@hanmail.net; 3 Department of Pediatric Dentistry, School of Dentistry, Kyung Hee University, Seoul 02447, Korea; pedochae@gmail.com

**Keywords:** dental caries, caries risk, saliva, significant caries index

## Abstract

This study aimed to investigate the salivary characteristics and individual daily living patterns in Korean children aged 12 years and evaluate their relationships according to the significant caries (SiC) index. The study sample consisted of 52 healthy Korean children. The subjects were allocated into a low caries-affected (low CA) group and a high caries-affected (high CA) group, according to the SiC index. Children underwent a standardized oral examination, and parents completed the questionnaires. Stimulated salivary samples were collected to evaluate the salivary pH, salivary flow rate, and salivary levels of *Mutans streptococci* (MS) and *Lactobacilli* (LB). The low CA group did not significantly differ from the high CA group for salivary flow rate and salivary pH. However, there were significant differences in salivary MS levels between the two groups (*p* < 0.05). Among the individual casual parameters, the prevalence of a sugar-associated primary energy source between meals was significantly higher in the high CA group than in the low CA group (*p* < 0.05). Within the limitations of this study, different levels of salivary MS and the consumption of different foods were observed in the low CA and high CA groups. The implications of these findings should be considered for caries susceptibility.

## 1. Introduction

Increasing public awareness of oral health care has led to a need for dental caries risk indicators to prevent and manage this disease [1,2]. Dental caries is the most prevalent infectious dental disease, which has been the primary cause of premature tooth loss in children [3,4]. The etiology of dental caries involves complex interactions between multifactorial predisposing factors [5]. Therefore, several caries risk assessment methods, including visual inspection and polymerase chain reaction, have been developed to identify more reliable predisposing factors [6,7]. Among the possible predisposing microbiological factors, *Mutans streptococci* (MS) and *Lactobacilli* (LB) are the primary bacteria associated with dental caries initiation. They can convert sucrose into acids, reduce oral pH, and survive below a pH level of 5.5 (threshold value for tooth demineralization) [8].

Salivary characteristics are also associated with the initiation and progression of dental caries [9,10]. In the previous literature, low salivary flow rate and poor buffering capacity have been suggested as dental caries-activity indicators [11,12]. However, the reliability of these factors in healthy children remains controversial. Previous literature evaluated children with specific conditions, such as asthma, chronic malnutrition, or children who received dental management under general anesthesia [12,13,14]. Other studies conducted on healthy patients evaluated salivary rate, pH, and salivary buffering capacity without analyzing their relationship with caries activity and caries experience [15].

Recently, individual casual parameters have emerged as major predisposing factors for the initiation and progression of dental caries. Evidence suggests that diet (particularly a sugar habit) can lead to dental caries [16,17]. Recent prospective studies show that feeding practices, the consumption of sugar-incorporated beverages, and other socio-behavioral factors can be potential risk factors for dental caries in children [18,19,20]. Dental caries is the result of complex interactions between various predisposing factors. Therefore, early detection and prediction are beneficial in the management and prevention of dental caries. Determining reliable risk predictors is a practical approach to preventing dental caries. This study aimed to investigate the salivary characteristics and individual daily living patterns in Korean children aged 12 years and evaluate their influence on dental caries experience.

## 2. Materials and Methods

### 2.1. Subject Enrollment and Sample Collection

This study was conducted according to the guidelines of the Declaration of Helsinki and approved by the Ethics Committee of Jeonbuk National University (ID: 201501004). Fifty-seven healthy children (aged 12 years) in two classes in one elementary school near Chonbuk National University were recruited as study subjects. Of these, 4 students were excluded from data as they moved to another school and could not participate in the study. One student was excluded from the data set due to uncooperative behavior. Finally, 52 children were selected (26 boys and 26 girls). The children were instructed to abstain from any oral hygiene for 24 h before sample collection and directed to brush their teeth after breakfast, 2 h before the appointment. After tooth brushing, no food or drink was allowed until saliva sampling. Stimulated saliva samples (at least 3 mL) were collected from all children 2 h after tooth brushing by asking them to chew on a piece of paraffin wax and spit into a 50 mL sterile tube. The samples were kept on ice and immediately transferred to the laboratory. Salivary tests and microbial analysis were performed on the same day.

One pediatric dentist performed the intraoral examination using a plane mouth mirror, following the World Health Organization (WHO) recommendations for oral epidemiological surveys. The DMFT/dmft index was evaluated (D/d = decay, M/m = missing, F/f = filling, t/T = teeth, D/d = DMFT/dmft).

### 2.2. Questionnaire for Individual Casual Parameters

A self-reported questionnaire was used to assess individual casual parameters in the school environment before the oral examination. The questionnaire included the following 4 questions:

- How many times do you brush your teeth in a day? (<3 times a day, ≥3 times a day)
- How long does it take you to clean your teeth after a meal? (<3 min, ≤10 min, and >10 min)
- How long do you brush your teeth for? (<3 min, ≥3 min)
- What is your primary source of energy between meals? (chocolates, candies, snacks, fruits, or vegetables)

Regarding primary food intake between meals, we divided this into two categories: sugar-intake habit (chocolates, candies, and snacks) and no sugar-intake habit (fruits and vegetables).

### 2.3. Defining Experimental Groups

In the present study, we allocated the subjects to two experimental groups according to the significant caries index (SiC index) with some modifications [21]. We calculated the DMFT/dmft values as the sum of DMFT and dmft value in each participant. We calculated the sum of all the DMFT/dmft values and divided it by the total number of participants. Finally, the mean DMTF/dmft index (mean value = 2.73) was used as the modified SiC index. Experimental groups were as follows:

(i) Low CA (low caries-affected) group: participants presented a DMFT/dmft index below the modified SiC index.
(ii) High CA (high caries-affected) group: participants presented a DMFT/dmft index above the modified SiC index.

### 2.4. Salivary Flow Rate and Salivary pH

All participants were instructed to chew on a piece of wax to stimulate salivary flow. After 30 s, they expectorated the saliva into a 50 mL tube for 5 min. After the disappearance of the salivary froth, the secretion rate was estimated in mL per min.

The pH values of saliva samples were estimated with a colorimetric kit (Saliva-Check Buffer, GC EUROPE N.V., Leuven, Belgium), according to the manufacturer’s instructions. In brief, a pH strip was placed into the collected sample of resting saliva for 10 s. The color of the strip was compared to the testing chart.

### 2.5. Microbiological Analysis of Saliva

A caries risk test (CRT bacteria, Ivoclar Vivadent AG, Liechtenstein) was used to measure the levels of MS and LB. The samples were vortexed for 10 sec. A portion of the saliva was pipetted onto two agar plates, one for MS and one for LB. The plates were incubated for 48 h at 37 °C. The number of colony-forming units (CFU) per mL of saliva was calculated according to the reference model chart provided in the instruction manual. The manual indicated the levels of bacteria as “low” (<10^5^ CFU/mL) or “high” (≥10^5^ CFU/mL). We assigned scores from 0 to 2. First, we divided the low-level mark into two scores: 0 (when there were no or almost no CFUs) and 1 (<10^5^ CFU/mL). The high-level mark corresponded to 2 (≥10^5^ CFU/mL).

### 2.6. Statistical Analysis

Data were analyzed using the Statistical Package for the Social Sciences (SPSS) 12.0 software (SPSS Inc., Chicago, IL, USA) for statistical analysis. The Shapiro–Wilk test was used to confirm that data were not normally distributed. All variables were statistically evaluated using either the Mann–Whitney test or Chi-square test. Correlations among numerical variables were analyzed using Spearman’s correlation test. A *p*-value of <0.05 was considered statistically significant.

## 3. Results

**Figure 1:**
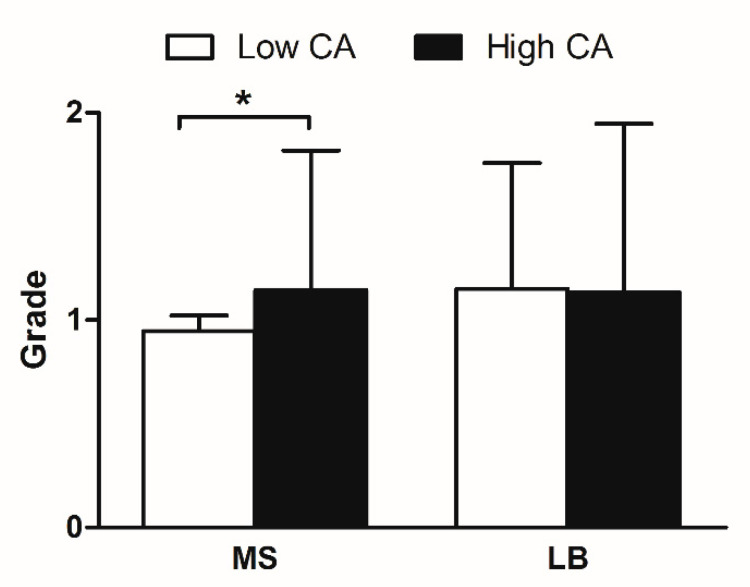
Presentation of microbiological test. Salivary levels of *Mutans streptococci* (MS) and *Lactobacilli* (LB) were recorded as follows; 0 = no or almost no CFUs, 1 = <10^5^ CFU/mL, and 2 = ≥10^5^ CFU/mL. Low CA; low caries-affected, High CA; High caries-affected * = statistically significant difference between the low CA and the high CA group (Mann–Whitney test, *p* < 0.05)

Of the 52 child participants, the low CA group consisted of 31 children, and the high CA group consisted of 21 children. Table 1 compares the values of salivary parameters between the two groups. The low CA group showed a mean salivary pH of 6.75 ± 0.49 and a mean saliva flow rate of 0.64 ± 0.44; the high CA group showed a mean salivary pH of 6.65 ± 0.57 and a mean salivary flow rate of 0.73 ± 0.36. There were no significant differences in salivary markers between the two groups. Salivary microbiological tests showed that the salivary levels of MS in the high CA group were statistically higher than those in the low CA group (*p* < 0.05). However, there was no statistical difference in salivary levels of LB between the two groups (Figure 1).

Table 2 and Table 3 show the correlations among all salivary parameters evaluated for the low CA group and the high CA group, respectively. A positive correlation between salivary MS levels and salivary LB levels was found in both the low CA group and the high CA group.

Table 4 shows comparisons of individual casual parameters between the low CA group and the high CA group. Among the individual casual parameters, a sugar-associated primary energy source consumed between meals was significantly higher in the high CA group (*p* < 0.05).

## 4. Discussion

In the present study, salivary parameters and individual casual parameters according to the SiC index were evaluated in 12-year-old Korean healthy children. Dental caries is the consequence of shifts in the balance between pathologic and protective factors [22]. Generally, dental caries is caused by organic acids produced by oral bacteria involved in the fermentation of dietary carbohydrates [23]. Additionally, biophysicochemical characteristics of saliva are considered important protective factors [24]. Therefore, analysis of oral bacteria and salivary characteristics can aid the early diagnosis and management of dental caries.

Saliva has an important role in oral health maintenance. It contributes to the oral defense system and shows buffering ability, antimicrobial activity, and calcium phosphate delivery to protect teeth from pathological factors [15]. Previous literature demonstrates that low salivary flow rate and low salivary pH are responsible for high caries prevalence [25]. Moreover, a strong association of salivary flow rate and salivary pH with dental caries has been confirmed in studies performed on subjects with specific medical conditions, suggesting that medications may considerably reduce the salivary flow rate and affect caries risk in patients [26]. However, the findings of the present study are in contrast with the results obtained from previous studies. This comparison may have been affected by sample characteristics. The results could be affected as the present research recruited only healthy children. A similar study conducted in Mexican schoolchildren demonstrated that salivary flow rate did not help predict caries risk [27]. Within the limitations of the present study, salivary flow rate and salivary pH did not significantly affect the initiation and progression of dental caries, indicating the presence of other markers that influence the initiation and progression of dental caries.

Microorganisms play an important role in the initiation and progression of dental caries as constituents of dental plaque biofilms [28]. A previous study on salivary MS levels suggested that the early acquisition of MS is related to a high incidence of early childhood dental caries and future caries susceptibility [29]. We found that 12-year-old healthy Korean children with high salivary MS levels had significantly high DMFT/dmft index values, a finding which is consistent with the results of previous studies. Salivary LB levels are also considered potent contributors to dental caries progression [30]. However, increased salivary LB levels were not found in the high CA group when compared to the low CA group in the present study. This finding is consistent with several studies showing that LB levels had a limited relationship to caries incidence [31,32]. Otherwise, in the present study, salivary LB levels showed a positive correlation with salivary MS levels (Table 2 and Table 3), regardless of caries experience. Thus, salivary MS levels demonstrate a strong correlation with dental caries initiation and progression. Further, this finding supports the hypothesis that salivary LB levels affect dental caries initiation and progression as secondary invaders [33].

Healthy eating patterns and meticulous tooth brushing are associated with oral health maintenance [34,35]. In the present study, the frequency and duration of tooth brushing and lag time between meals and tooth brushing did not differ between the low CA group and the high CA group. Only the primary energy source between meals was significantly associated with previous caries experiences, a finding that correlates with previous studies. A direct relation between sugar intake and dental caries has been reported, as cariogenic bacteria grow in the presence of fermentable carbohydrates [36]. Previous literature suggested that higher chocolate and candy consumption are risk factors for dental caries development in both primary and permanent dentition, as they remain on the tooth surface for hours without providing any nutritional value [37,38,39].

The present study has several limitations. The study was cross-sectional in design with a small sample size affecting the generalizability of the results. However, difficulties in participant enrollment existed as several parents whose children were requested to participate in this study did not consent. Further, some children who consented initially refused to provide salivary samples. Moreover, the assessment of individual casual parameters was based on a self-reported questionnaire. Thus, the overestimation or underestimation of these parameters could be possible. Future studies with a larger sample size are required.

## 5. Conclusions

Dental caries experiences in 12-year-old healthy Korean children showed a significant association with salivary MS levels and dietary sugar intake. The results indicate that these parameters could be reliable predictive indicators of dental caries in healthy Korean children aged 12 years. Incorporating these parameters in tests conducted during annual in-school oral examination is also recommended. This approach could achieve the prediction and early detection of dental caries and improve oral health status in children.

## Figures and Tables

**Table 1:** Comparison of salivary pH and salivary flow rate according to the significant caries index.

Salivary Parameters	Low CA ^1^	High CA ^2^	*p*-Value
Salivary pH	6.75 ± 0.49	6.65 ± 0.57	0.651
Saliva flow rate	0.64 ± 0.44	0.73 ± 0.36	0.112

^1^ Low CA = low caries-affected, ^2^ high CA = high caries-affected.

**Table 2:** Correlation coefficients of caries parameters in the low caries-affected group.

Salivary Parameters	Salivary pH	Salivary Flow Rate	MS ^1^	LB ^2^
Salivary pH	1			
Salivary flow rate	0.288	1		
MS	−0.326	−0.214	1	
LB	−0.195	0.323	0.370 *	1

^1^ MS = *Mutans streptococci*, ^2^ LB = *Lactobacilli*, * = statistically significant difference between corresponding parameters (Spearman’s correlation test, *p* < 0.05).

**Table 3:** Correlation coefficients of caries parameters in the high caries-affected group.

Salivary Parameters	Salivary pH	Salivary Flow Rate	MS ^1^	LB ^2^
Salivary pH	1			
Salivary flow rate	0.265	1		
MS	−0.033	−0.055	1	
LB	−0.357	0.150	0.482 *	1

^1^ MS = *Mutans streptococci*, ^2^ LB = *Lactobacilli*, * = statistically significant difference between corresponding parameters (Spearman’s correlation test, *p* < 0.05).

**Table 4:** Comparison of individual casual parameters by the significant caries index.

Individual Casual Parameters	Low CA ^1^	High CA ^2^	*p*-Value
Frequency of tooth brushing			
<3 times a day	9 (29%)	6 (28.6%)	0.971
≥3 times a day	22 (71%)	15 (71.4%)	
Lag time between meals and tooth brushing			
<3 min	13 (41.9%)	7 (33.3%)	0.435
<10 min	8 (25.8%)	9 (42.9%)	
≥10 min	10 (32.3%)	5 (23.8%)	
Tooth brushing duration			
<3 min	19 (61.3%)	13 (61.9%)	0.964
≥3 min	12 (38.7%)	8 (38.1%)	
Primary source of energy between meals			
Sugar intake	19 (61.3%)	19 (90.5%)	0.02 *
No sugar intake	12 (38.7%)	2 (9.5%)	

^1^ Low CA = low caries-affected, ^2^ high CA = high caries-affected, * = statistically significant difference between low CA and high CA groups (Chi-square test).

## Data Availability

All relevant data are within the paper.
